# Draft *De Novo* Transcriptome of the Rat Kangaroo *Potorous tridactylus* as a Tool for Cell Biology

**DOI:** 10.1371/journal.pone.0134738

**Published:** 2015-08-07

**Authors:** Dylan B. Udy, Mark Voorhies, Patricia P. Chan, Todd M. Lowe, Sophie Dumont

**Affiliations:** 1 Department of Cell & Tissue Biology, University of California San Francisco, San Francisco, CA, United States of America; 2 Department of Microbiology & Immunology, University of California San Francisco, San Francisco, CA, United States of America; 3 Maverix Biomics, Inc., San Mateo, CA, United States of America; 4 Department of Biomolecular Engineering, University of California Santa Cruz, Santa Cruz, CA, United States of America; 5 Department of Cellular & Molecular Pharmacology, University of California San Francisco, San Francisco, CA, United States of America; Stanford University, UNITED STATES

## Abstract

The rat kangaroo (long-nosed potoroo, *Potorous tridactylus*) is a marsupial native to Australia. Cultured rat kangaroo kidney epithelial cells (PtK) are commonly used to study cell biological processes. These mammalian cells are large, adherent, and flat, and contain large and few chromosomes—and are thus ideal for imaging intra-cellular dynamics such as those of mitosis. Despite this, neither the rat kangaroo genome nor transcriptome have been sequenced, creating a challenge for probing the molecular basis of these cellular dynamics. Here, we present the sequencing, assembly and annotation of the draft rat kangaroo *de novo* transcriptome. We sequenced 679 million reads that mapped to 347,323 Trinity transcripts and 20,079 Unigenes. We present statistics emerging from transcriptome-wide analyses, and analyses suggesting that the transcriptome covers full-length sequences of most genes, many with multiple isoforms. We also validate our findings with a proof-of-concept gene knockdown experiment. We expect that this high quality transcriptome will make rat kangaroo cells a more tractable system for linking molecular-scale function and cellular-scale dynamics.

## Introduction

For the last half-century, epithelial cells from the long-nosed potoroo (*Potorous tridactylus*, a marsupial known as the rat kangaroo) have been a commonly used system for studying cellular-scale dynamics. The rat kangaroo has only five pairs of autosomes and three sex chromosomes (in males) [1], making it well suited for cell culture studies of chromosomes. Indeed, marsupials have among the lowest numbers of chromosomes among mammals [2]. The PtK2 cell line was derived from male rat kangaroo kidney epithelial cells in 1962 [3], and PtK cell lines (PtK1, from female, and PtK2, from male) have since become broadly used to study how mammalian cells segregate their chromosomes during cell division. PtK cells are well suited for visualizing the cellular dynamics of mitosis through microscopy: i) they possess large chromosomes that are few in number, ii) they are large, adherent and flat, and iii) they can remain relatively flat throughout mitosis—in contrast to many other mammalian cells [4]. Because of the ease of imaging, PtK cells have also been broadly used to probe dynamics of processes such cell migration, cell adhesion and protein trafficking.

However, probing the molecular basis of these cellular processes in PtK cells has been challenging due to the absence of rat kangaroo gene sequence information. We list four example reasons. First, while RNAi is possible in PtK cells, siRNA design requires sequencing the target gene, and cannot minimize for off-target effects because the sequences of other genes are not known. Only three rat kangaroo genes have so far been knocked down by RNAi (*KIF2C/MCAK*, *KIF11/EG5*, *NDC80/HEC1*) [5,6], based on genes sequenced by individual laboratories (the first fully, others partially). In PtK cells, RNAi as well as endogenous gene knockout or editing (e.g. CRISPR-based) would be facilitated by broadly available gene sequences. Second, while expression of exogenous (e.g. mutated, or fluorescently-tagged) genes is possible in PtK cells, these exogenous genes have come from other species, which can lead to experiment interpretation artifacts due to cross-species differences in sequence and function. Third, many antibodies developed against proteins from other species do not work in rat kangaroo. Fourth, modern genome- or transcriptome-wide tools such as gene expression profiling are currently out of reach for rat kangaroo cells. As of June 2015, the NCBI database has nucleotide sequences for only 142 rat kangaroo genes (many for the same genes), with only one of them (*KIF2C/MCAK*) being thought to be a key mitotic gene. While the genomes of some marsupials less commonly used in cell biology have been sequenced (gray short-tailed opossum [7], Tasmanian devil [8], and tammar wallaby [9]), that of the rat kangaroo has not.

Here, we present the draft *de novo* assembly of the rat kangaroo transcriptome, which provides the gene sequence information necessary to make possible i) molecular-scale perturbations (such as gene knockdown, knockout and editing) and molecular readouts (such as endogenous gene fluorescent tagging), and ii) relative gene expression abundance analyses. We performed high-throughput sequencing, assembly and annotation of this draft transcriptome based on PtK2 cell transcripts. Based on an analysis of a subset of genes, we expect that full-length sequences are available for most genes, and that the database contains multiple transcript isoforms for many genes. Finally, we performed an experimental test that helps validate the rat kangaroo transcriptome, and its usability for siRNA design and gene knockdown. We expect that this high quality transcriptome will make rat kangaroo cells a more tractable system for mechanistic experiments linking molecular-scale function and cellular-scale dynamics, and for transcriptome-wide gene expression analyses.

## Results and Discussion

### Rat kangaroo transcriptome sequencing, assembly and annotation

To sequence the rat kangaroo transcriptome, we extracted total RNA from unsynchronized cultured rat kangaroo PtK2 cells. Thus, this transcriptome reflects transcripts present in these cultured PtK2 kidney epithelial cells. We enriched for mRNA using poly(A) tail selection and constructed a cDNA sequencing library with average insert size of 275 bp. We performed next-generation sequencing via a paired-end 150-cycle rapid run on the Illumina HiSeq2500, generating 679,303,792 raw reads (Table 1), corresponding to very high coverage depth. We sequenced over 99 billion nucleotides, and these had a Q20 (i.e. sequencing error rate <1%) of 98.4% and GC content of 49.9% (Table 1).

**Table 1:** Rat kangaroo transcriptome-wide statistics.

Total raw reads	679,303,792
Total clean reads	678,793,914
Total nucleotides	99,012,349,450
Q20 percentage	98.4%
GC percentage	49.9%
Mean length of Trinity transcripts	1,197
N50 of Trinity transcripts	3,405
Total Trinity transcripts assembled	347,323
Trinity transcripts without open reading frames	272,033
Trinity transcripts with open reading frames	75,290
Total Unigenes	252,022
Unigenes without open reading frames	231,943
Unigenes with open reading frames	20,079
Distinct protein coding clusters	7,846
Distinct protein coding singletons	12,233
Core ribosomal proteins with open reading frames (of 75)	65
Core ribosomal proteins with assembled transcripts (of 75)	75
Completely mapped CEGMA core eukaryotic genes (of 248)	239
Partially mapped CEGMA core eukaryotic genes (of 248)	248

**Fig 1:**
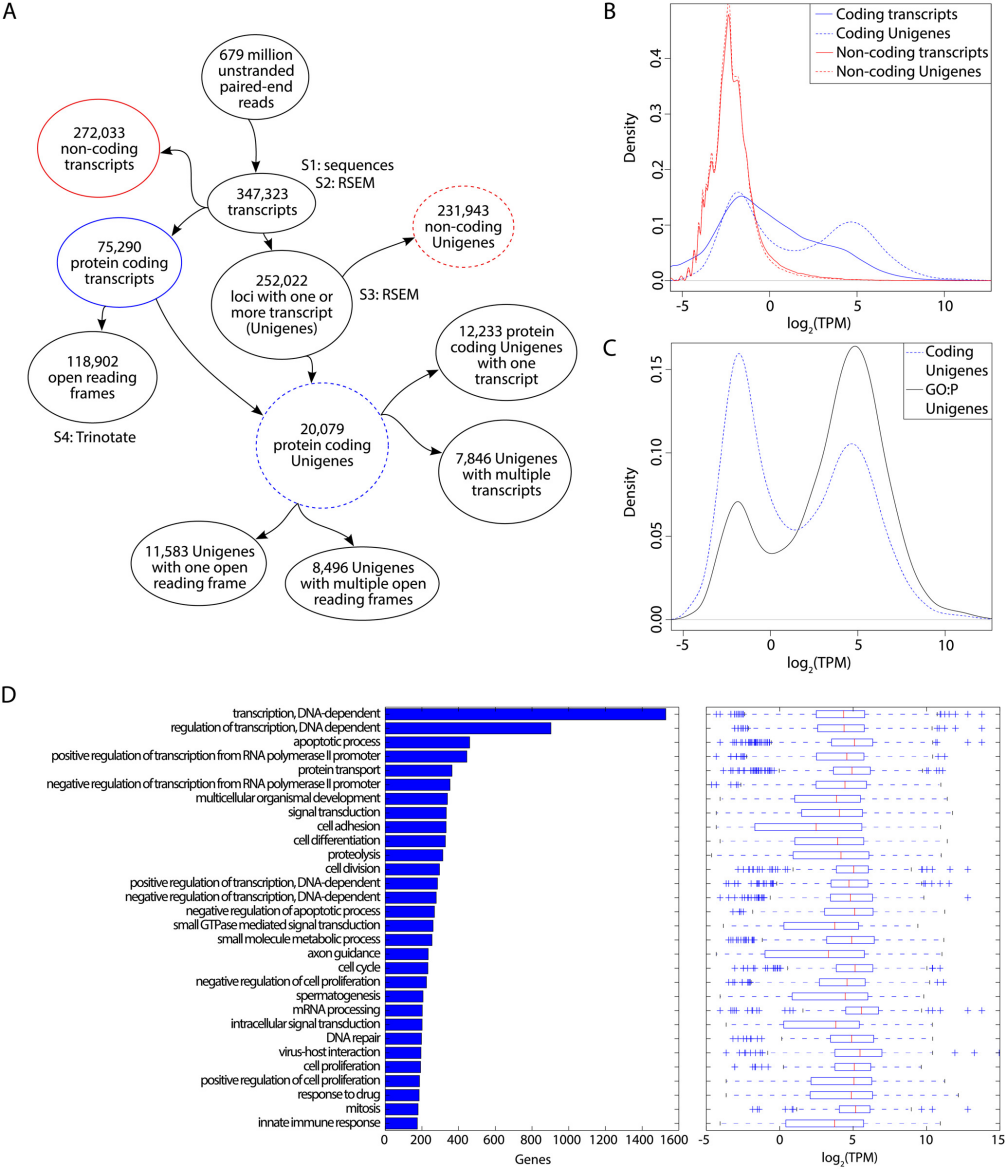
Rat kangaroo transcriptome-wide analysis. **(A)** Flow chart indicating the numbers of and relationships among coding and non-coding transcripts and Unigenes. Labels S1-S4 indicate from which datasets Supporting Information S1–S4 files arise. **(B)** Distributions of RSEM-inferred TPM values for: all protein coding (blue) or non-coding (red) transcripts (solid) or Unigenes (dashed) in the rat kangaroo transcriptome. These colors and line styles are consistently used in (A). **(C)** Distributions of protein coding Unigene TPMs (as in (B)) and the subset of protein coding Unigenes annotated by GO biological process terms (black solid). **(D)** The 30 GO biological process terms occurring most frequently in the annotation of the 20,079 Unigenes are listed with number of Unigenes annotated by each term (left) and distribution of RSEM gene-level abundance estimates for the annotated Unigenes (right; boxes indicate interquartile range (IQR), red bars indicate medians, whiskers indicate most extreme data points within 1.5*IQR of the boxes, and crosses indicate outliers).

We assembled the transcriptome *de novo* using the Trinity software package [10,11]. This software was specifically designed for reconstructing a full-length transcriptome from RNA sequencing (RNA-Seq) data when a genome sequence is not available. From this point on, we will refer to our assembled transcript isoforms as “Trinity transcripts” and to inferred loci emitting one or more related isoforms as Unigenes. The breakdown of Trinity transcripts and Unigenes with respect to coding potential and isoform multiplicity is given in Fig 1A. We assembled 347,323 different Trinity transcripts (S1 File), and these had a mean length of 1,197 nt and N50 of 3,405 nt (i.e. 50% of the assembled bases were incorporated in Trinity transcripts of ≥3,405 nt; Table 1). We analyzed the relative abundance of each Trinity transcript (S2 File) and Unigene (S3 File), reported as TPM (transcripts per million; Fig 1B), using RSEM (RNA-Seq by Expectation Maximization) [12]. There was a relatively high number of non-coding Unigenes with predominantly low abundance and low isoform multiplicity (Fig 1B). In contrast, the 20,079 protein coding Unigenes had an average of 3.7 isoforms each and displayed a bimodal abundance distribution, with about 10,000 Unigenes at a low abundance similar to non-coding Unigenes, and a second population of about 10,000 higher abundance Unigenes (Fig 1B).

We annotated the translated Trinity transcripts using i) BLASTP [13] similarity search against the SwissProt protein database [14], ii) protein family classification based on the PFAM database [15], iii) gene ontology (GO) [16] mapping, and iv) orthologous groups of gene (eggNOG, evolutionary genealogy of genes: Non-supervised Orthologous Groups) classification [17] (S4 File). In total, the 75,290 Trinity transcripts that were identified with open reading frames correspond to 20,079 Unigenes (unique genes), of which 7,846 have transcripts in a distinct cluster and 12,233 have a single transcript not in a cluster (Table 1). As an initial test of transcriptome quality, we searched in the transcriptome for the mitotic gene *KIF2C/MCAK* (Table 2 gene marked with a star), whose full transcript sequence was previously known from PtK1 cells [5], and available on NCBI. There was only one nucleotide mismatch between both protein coding sequences, consistent with accurate transcriptome sequencing, assembly and annotation.

**Table 2:** Transcript-level analysis for 25 cell division genes.

Cellular process	Gene symbol	Length of protein in amino acids: rat kangaroo/human^1^	TPM in rat kangaraoo transcripttome^2^	Rat kangaroo protein sequence identity (%) compared to ortholog in:			# of full-length rat kangaroo transcript isoforms
				Gray short-tailed opossum *(M*. *Domestica)*	Tasmanian devil *(S*. *harrisii)*	Human *(H*. *sapiens)*	
Cytokinesis	*PRC1*	618/620 (99.7%)	73.2	89	89	79	3
	*ANLN*	1126/1142 (98.6%)	186.8	74	69	67	2
Kinetochore (structure, signaling)	*NDC80*	646/642 (100.6%)	26.9	81	84	72	2
	*NUF2*	460/464 (99.1%)	25.7	80	86	68	1
	*SPC24*	197/197 (100%)	44.8	79	74	64	1
	*SPC25*	238/224 (106.3%)	17.5	86	66	67	1
	*CENPC*	996/943 (105.6)	7.2	69	72	38	8
	*CENPT*	501/558 (89.8%)	16.6	72	76	46	2
	*SKA1*	253/255 (99.2%)	5.7	88	89	66	1
	*MIS12*	203/205 (99.0%)	19.9	79	No match**	58	1
	*CASC5*	2055/2342 (87.7%)	3.3	66	78	47	2
	*CDC20*	499/499 (100%)	76.9	98	98	90	1
	*AURKB*	349/344 (101.5%)	62.7	86	89	76	2
	*AURKA*	405/403 (100.5%)	23.7	92	91	76	2
Microtubule cytoskeleton	*KIF2C* *	728/725 (100.4%)	27.9	92	94	81	4
	*KIF11*	1053/1056 (99.7%)	20.3	88	89	72	1
	*DYNC1H1*	4648/4646 (~100%)	82.2	99	99	98	1
	*DCTN1*	1243/1246 (99.8%)	63.8	97	97	91	1
	*MYL9*	172/172 (100%)	274.6	100	100	99	1
	*MAPRE1*	268/268 (100%)	51.7	98	93	92	1
	*CLASP1*	1462/1471 (99.4%)	7.5	98	99	95	2
	*TUBB4B*	445/445 (100%)	1232.0	100	100	100	1
Chromosome maintenance	*SMC1A*	1233/1233 (100%)	72.2	99	99	97	1
	*SMC3*	1217/1217 (100%)	59.8	99	100	99	1
	*SMC2*	1197/1197 (100%)	12.1	94	95	85	1

^1^ Length (amino acids) of most abundant, full-length rat kangaroo protein (numerator) compared to the canonical human protein length (denominator). This protein sequence is also used for sequence identity comparisons to orthologs further in the table.

^2^ TPM values in the table correspond to the most abundant, full-length rat kangaroo Trinity transcript for each gene.

* As noted earlier, rat kangaroo *KIF2C* was previously sequenced [5] (NCBI DQ444242.1). This published sequence differs by only one amino acid from the *KIF2C* sequence in this transcriptome.

** No orthologous gene annotated in *S*. *harrisii*.

### Rat kangaroo transcriptome data sharing

We have made sequencing, assembly and annotation of data publicly available as a resource for the community. First, all raw sequencing reads have been submitted to the NCBI Sequence Read Archive (SRA; http://www.ncbi.nlm.nih.gov/sra) under accession number SRP055986. Second, as noted above we have included processed data as Supporting Information files: Trinity assembled transcript sequences (S1 File), isoform abundances (S2 File), Unigene abundances (S3 File) and protein annotations (S4 File). Third, we created a web portal (http://dumontlab.ucsf.edu/ratkangaroo.htm) for the rat kangaroo transcriptome to allow researchers across fields to more easily access the assembled and annotated data. There, the first browser allows the user to browse different Trinity transcripts using a custom UCSC Genome Browser interface running in the Amazon Web Services (AWS) cloud and maintained by Maverix Biomics, Inc., where mRNA transcripts have been substituted for chromosomes. Trinity transcripts are assembled and numbered, and a BLAT (BLAST-like alignment tool) [18] search tool enables mapping of any input protein or nucleotide sequences to the rat kangaroo transcriptome.

The second, custom-designed transcript browser contains Trinotate-annotated transcript information, and allows the user to search by gene description, or transcript ID (from the first browser). This browser contains the above annotation analyses, with links to external databases, and abundance (TPM, and FPKM, fragments per kilobase of transcript per million fragments mapped) statistics for each Trinity isoform. Both browsers were designed to be used by a broad range of biologists and do not require specialized knowledge.

### Functional classification of the rat kangaroo transcriptome

Of the 20,079 protein coding Unigenes, about half (9,858) are associated with at least one GO biological process (GO:P) annotation. The annotation frequency is bimodal with respect to transcript abundance (as estimated by the RSEM gene-level TPM values), with annotations present for about 3/4 (7,435) of the most abundant 10,000 Unigenes but only about 1/4 (2,423) of the remaining 10,079 Unigenes (Fig 1C). This suggests that better sampling of robustly expressed genes leads to more complete transcript assembly and, therefore, more correct inference of open reading frames and gene annotations. A similar abundance bias is evident for the Pfam, GO molecular function, and eggNOG annotations.

The 9,858 GO:P annotated Unigenes are associated with 7,032 unique GO:P terms, with the 10 most frequent terms spanning about 1/3 (3,439) and the 30 most frequent terms spanning about 1/2 (5,318) of the annotated Unigenes (Fig 1D). The top GO:P terms are dominated by regulatory processes, primarily regulation of transcription (including the most frequent term and, redundantly, five more specific terms), as well post-translational (primarily via protein kinases) and post-transcriptional (mRNA processing) modifications. In contrast, only one of the top terms annotates core metabolism. This is consistent with the eggNOG annotation, where almost twice as many genes are annotated by the "information storage and processing" and "cellular processes and signaling" functional classes relative to the "metabolism" functional classes. A few terms, notably "virus-host" interaction, are due in part to abundantly transcribed retroviruses.

### Transcript-level analysis for 25 cell division genes

To probe the depth and accuracy of the rat kangaroo transcriptome, we performed selected analyses on 25 chosen genes (Table 2). We chose 25 genes with functions important at mitosis, as this would also inform on the value of this transcriptome for mitosis research done in PtK cells. We selected genes involved in cytokinesis, kinetochore function, the microtubule cytoskeleton, and chromosome maintenance. All 25 genes we searched for were not only present in the transcriptome, but present with at least one (and often more) full-length Trinity transcript isoform—consistent with data above suggesting a deeply sequenced transcriptome with full-length transcripts of most genes expressed in PtK2 cells. The most abundant Trinity transcript of each gene had a TPM value ranging from 3 to more than 1,200. Finally, rat kangaroo Trinity transcripts typically had the highest sequence similarity to the gray short-tailed opossum (*Monodelphis domestica*) and Tasmanian devil *(Sarcophilus harrisii*) proteins when a BLASTP search was performed against all sequenced mammals. Proteins expected to be highly conserved were indeed highly conserved; for example, a tubulin [19] gene (TUBB4B; Table 2) is 100% conserved between rat kangaroo and human. As additional measures of transcriptome breadth, we asked if 75 core eukaryotic ribosomal proteins or 248 core eukaryotic genes (CEGs) could be found in rat kangaroo. We found transcripts for all 75 ribosomal proteins, of which 65 were present in the translated open reading frame set; likewise, CEGMA [20] identified complete orthologs for 239 CEGs and at least partial orthologs for all 248 CEGs (Table 1). Taken together, the data suggest the rat kangaroo transcriptome is of both high quality and high depth, and that it will be a valuable resource for probing the molecular basis of mitosis and other cellular processes.

### Proof-of-concept experimental validation of rat kangaroo transcriptome: single gene analysis (*PRC1*)

To probe and highlight the value of the rat kangaroo transcriptome for the study of an individual gene and its function, we focused on a selected gene that has not been perturbed in rat kangaroo cells before. We chose *PRC1* because i) it has a well-characterized function at mitosis, ii) reagents (antibodies) are available for it, and iii) loss of this gene results in a phenotype that can be easily scored at the single cell level by imaging. PRC1 participates in spindle midzone microtubule formation [21], and is essential for cytokinesis in mammals [21,22].

**Fig 2:**
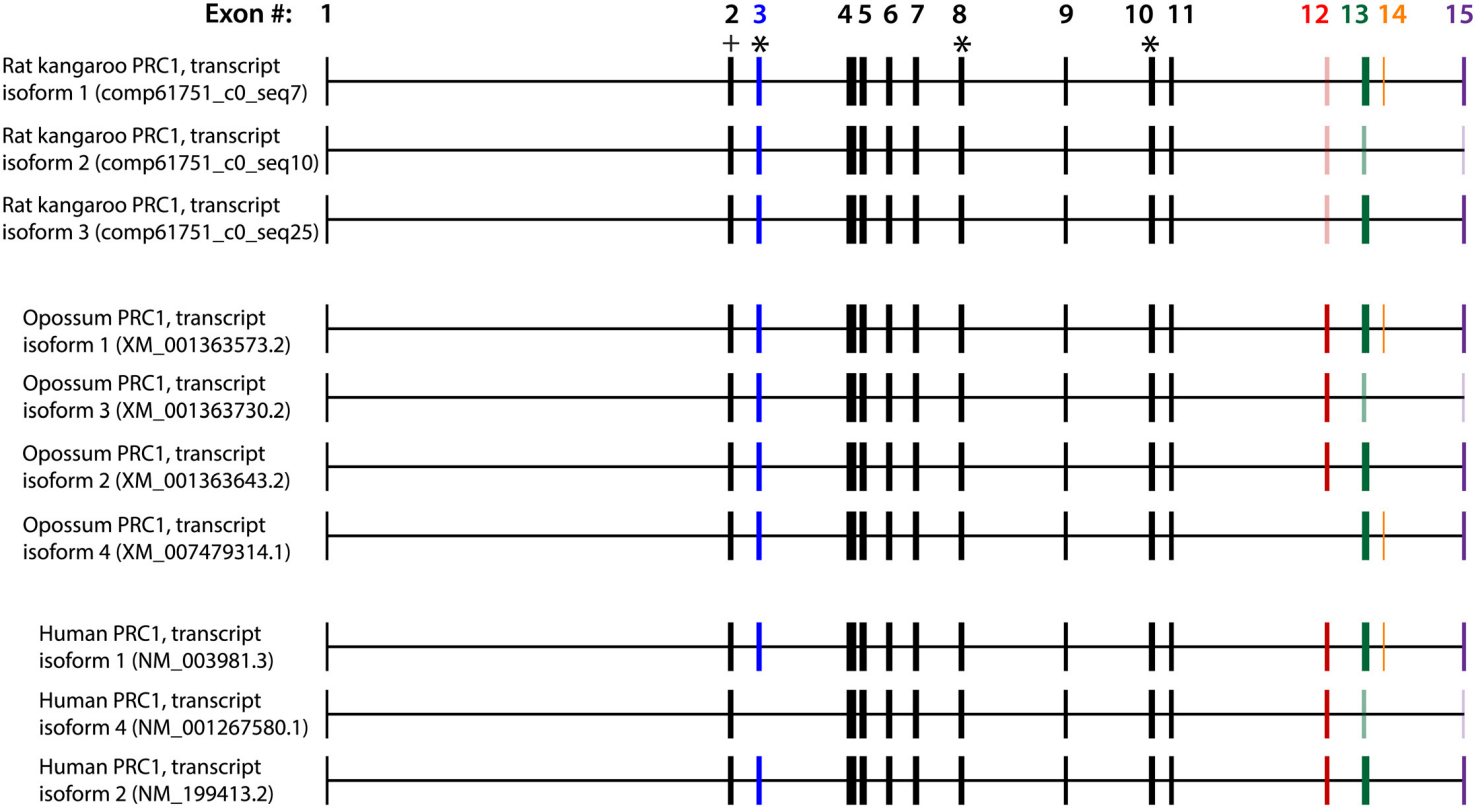
Single gene analysis (*PRC1*): Within- and cross-species transcript isoform comparisons. Alignment of all *PRC1* mRNA isoforms from rat kangaroo, gray short-tailed opossum, and human relative to the human genomic *PCR1* DNA sequence, providing a basis for within- and cross-species putative splicing isoform comparisons. The numbered boxes correspond to exons, while the horizontal lines in between exons correspond to introns. Exons that change in length or are absent within or between species are colored for emphasis, with darker colors marking longer exon isoforms within a given exon (length differences may be only a few amino acids). The target location of the chosen siRNA PRC1-A for the proof-of-concept experiment presented below is marked by “+”, and the target locations of other tested siRNAs are marked by “*”. While the overall sequence similarity is much higher between rat kangaroo and opossum than rat kangaroo and human, the inferred structures are highly concordant among all three species.

First, we aligned all three rat kangaroo full-length *PRC1* Trinity transcripts and *PRC1* transcript isoforms from the gray short-tailed opossum and human with the *PRC1* gene sequence from the human genome (Fig 2). This revealed putative splicing sites in rat kangaroo Trinity transcript isoforms. Of the putative exons inferred by this alignment, only #13–15 varied in length among the three rat kangaroo isoforms. No sequence differences were otherwise found among the three rat kangaroo transcripts. All putative exon lengths are conserved across species and isoforms except for exons #3 and #12–15, which can be absent or vary in length. The *PRC1* sequence is similar among species (Table 2), and all species express a similar number (three to four) of *PRC1* isoforms. Such sequence comparisons in other genes could be used to examine cross-species differences in protein functions.

**Fig 3:**
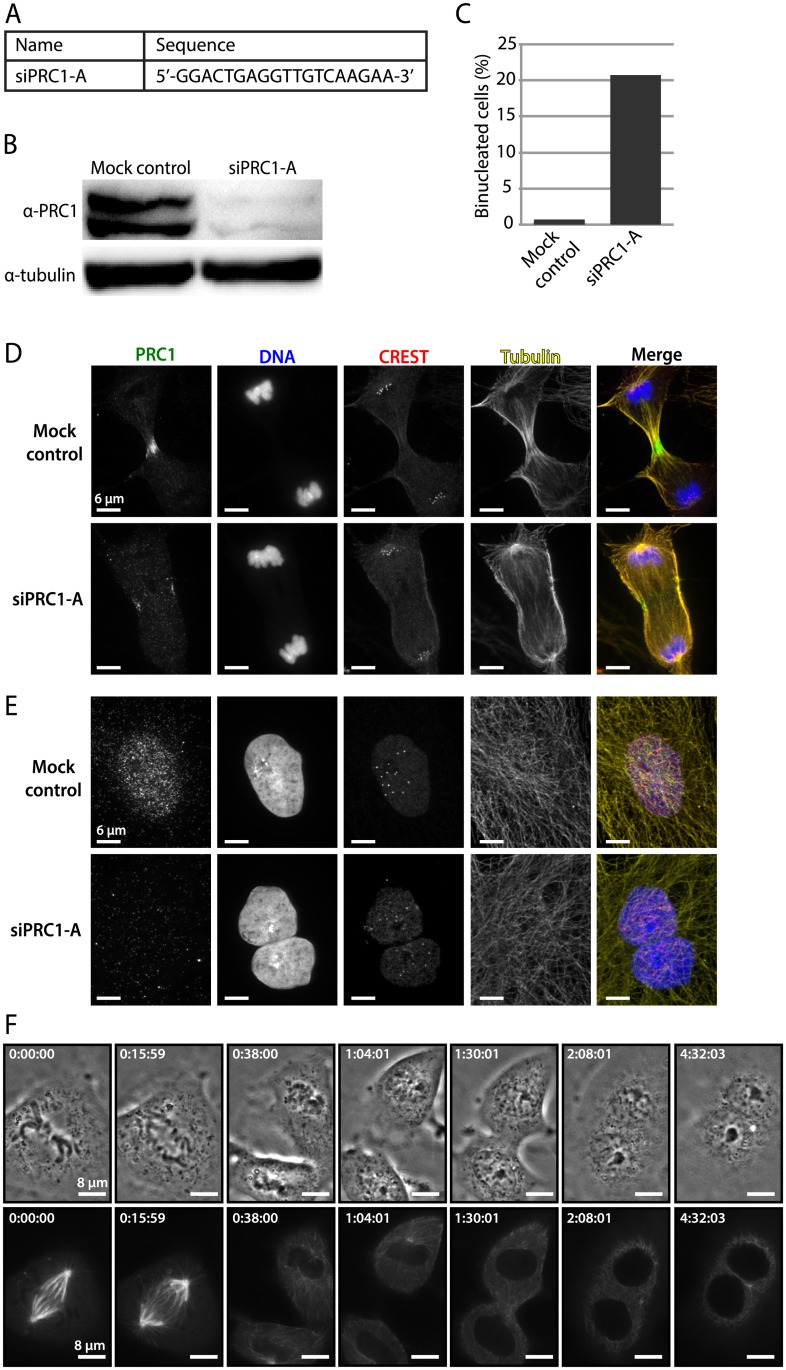
Proof-of-concept experimental validation of rat kangaroo transcriptome: single gene analysis (*PRC1*). **(A)** Sequence of the 19 bp siRNA (siPRC1-A) designed against the rat kangaroo *PRC1* sequence from the transcriptome. This siRNA was used in subsequent experiments to validate the siRNA design protocol and confirm successful knockdown. **(B)** Immunoblot demonstrating successful depletion of PRC1 protein after RNAi. Here and forward, “mock control” represents an Oligofectamine-only control. The tubulin band serves as a loading control. **(C)** Percentage of binucleated cells in a population of control and siRNA-treated cells 48 h after treatment. In the control population, 2/278 cells were binucleated (0.7%). In the siRNA-treated population, 53/256 cells were binucleated (20.7%). **(D)** Immunofluorescence image showing phenotype during cytokinesis in control and siRNA-treated cells. PRC1 localizes at the spindle midzone in control cells during cytokinesis, and is absent from the midzone during cytokinesis in siRNA-treated cells. **(E)** Immunofluorescence image showing the interphase localization of PRC1 in control and siRNA-treated cells. Control cells show nuclear localization of PRC1 and are mononucleated, while siRNA-treated cells show no PRC1 localization and are often binucleated. **(F)** Time strip showing cytokinesis failure for siRNA-treated cells. Phase contrast (top) and GFP-tubulin (bottom) images are shown. Cytokinesis appears to be successful at 1:04:01, but at 1:30:01 it becomes clear that the daughter cells never completely separated and they have come back together to form one binucleated cell. In total, 16 cells were imaged through cytokinesis in this manner, and four appeared to undergo this type of failed cytokinesis, a ratio similar to that revealed by counting of binucleated cells by immunofluorescence (C). Time in h:min:sec.

Second, we sought to experimentally validate the rat kangaroo transcriptome and determine how robust siRNA design would be given this high quality transcriptome. We did this proof-of-concept experiment by knocking down *PRC1* via RNAi in PtK2 cells. We initially designed four siRNA sequences against exons that were present in all three *PRC1* isoforms. We used three openly available siRNA design algorithms and, in order to limit off-target effects of the siRNAs, we chose the highest scoring siRNA suggestions that did not have related sequences in the rest of the transcriptome (see Methods). All four siRNAs led to an increase in binucleated cells, suggesting that cytokinesis failed due to successful knockdown of *PRC1*. For subsequent experiments, we used the siRNA (siPRC1-A, Figs 2 and 3A) that led to the largest increase in binucleated cells. This siRNA led to an 84% loss in PRC1 protein expression at the cell population level as determined by immunoblot (Fig 3B). Imaging revealed that 20% of cells treated with this siRNA were binucleated (Fig 3C), unable to complete cytokinesis. The latter is similar to previous observations reported in human cells where *PRC1* RNAi knockdown led to 23% binucleated HeLa cells [21]. In addition, immunofluorescence revealed a marked reduction in PRC1 expression at both mitosis (Fig 3D) and interphase (Fig 3E), and defects in furrow ingression at cytokinesis (Fig 3D). Live imaging of PtK2 cells suggested that in some cases the furrow begins to ingress, but regresses after cytokinesis failure, leading to binucleation (Fig 3F), consistent with a prior report in HeLa cells [21]. Thus, the rat kangaroo transcriptome facilitates siRNA design, and *PRC1* RNAi phenotypes parallel previously reported ones, demonstrating the correct sequence, assembly and annotation of *PRC1* in this transcriptome.

## Conclusions

In summary, we provide the first rat kangaroo transcriptome, and provide analysis and experiments indicating that it is a high quality (depth, breadth, and accuracy), validated resource that can be used to learn new biology. The transcriptome will make rat kangaroo cells more amenable to both older and newer molecular-based tools such as gene knockdown or knockout, mutational analysis or fluorescence-tagging of native genes, antibody generation, transcriptome-wide expression profiling, and phylogenetic analysis. We expect the transcriptome to make rat kangaroo cells a good system not only for imaging cellular dynamics, but also for probing the molecular basis of these dynamics.

## Materials and Methods

### Rat kangaroo PtK2 cell RNA extraction

PtK2 cells were plated at 30% confluency on a 10 cm tissue culture dish and grown unsynchronized at 37°C and 5% CO2 for 48 h. RNA was extracted and purified using the RNeasy Midi Kit (Qiagen #75142, Venlo, Limburg, Netherlands). Cell culture media was aspirated out of the dish and 1 mL of Buffer RLT (with β-ME) was added to lyse cells. Lysate was collected using cell lifters (Corning, Corning, New York), vortexed for 10 sec, and passed through a 27-gauge needle 10 times to homogenize cells. The purification continued according to the manufacturer’s protocol. The PtK2 cell line [3] was obtained from the lab of Ted Salmon in March 2007.

### Rat kangaroo cDNA library construction and sequencing

Total RNA quality was assessed by spectrophotometer (NanoDrop, Thermo Fisher Scientific Inc., Waltham, MA) and the Agilent 2100 Bioanalyzer (Agilent Technologies, Santa Clara, CA). mRNA was purified from 1 μg of total RNA using poly(A) tail selection. RNA sequencing libraries were generated using the TruSeq RNA Sample Prep Kit (Illumina, San Diego, CA) with multiplexing primers, according to the manufacturer’s protocol. The cDNA library was constructed with average inserts of 275 bp, with non-stranded library preparation. Fragment size distributions were assessed using the Bioanalyzer 2100 and the DNA high-sensitivity chip. Library concentrations were measured using the KAPA Library Quantification Kit (Kapa Biosystems Inc., Wilmington, MA) and run on the Illumina HiSeq2500. Sequencing was performed via a paired-end 150 cycle rapid run on 2 lanes of the Illumina HiSeq2500, generating more than 300 million pairs of reads as desired.

### Rat kangaroo transcriptome assembly, abundance and annotation

Raw sequencing reads generated for the cDNA library of the rat kangaroo cell line (PtK2) were quality checked for potential sequencing issues and contaminants using FastQC v0.10.1 [23], invoked as “fastqc—nogroup-t 20”. Adapter sequences, primers, Ns, and reads with quality score below 28 were trimmed using fastq-mcf of ea-utils 1.1.2–537 [24], invoked as “fastq-mcf-k 0-q 0-l 20”and PRINSEQ 0.20.3 [25], invoked as “prinseq-lite.pl-trim_ns_right 2-trim_qual_left 28-trim_qual_right 28-min_qual_mean 28-min_len 20”. Reads with a remaining length of fewer than 20bp after trimming were discarded. *In silico* normalization using Trinity utility [10] was performed on the post-trimmed paired-end reads to achieve a maximum of 30X coverage for assembly, *viz*. *“*normalize_by_kmer_coverage.pl—JM 300G —max_cov 30—JELLY_CPU 50—PARALLEL_STATS—pairs_together*”*. Normalized reads were used for *de novo* transcriptome assembly using Trinity r2013-02-25 [10], invoked as “Trinity.pl—JM 300G —CPU 50—bflyHeapSpaceMax 10G —bflyCalculateCPU—bflyGCThreads 2”, that produced the Trinity transcripts in FASTA file format (S1 File).

To test for the possibility of “jackpot” effects in library preparation, we calculated a library complexity metric [26]. Specifically, for each of the two sequencing lanes, a random sample of 2.5 million read pairs was mapped to the assembled transcriptome by bowtie2 2.0.0-beta6 in unique hits mode (-k1) using a maximum fragment length of 800 (-X 800). For concordantly mapped read pairs, we then calculated the unpaired (per-read) library complexity as the number of unique read start sites divided by the number of mapped reads (0.74 for both lanes) and the paired library complexity as the number of unique fragment start and stop sites divided by the number of mapped reads (0.98 for both lanes). From this, we conclude that there were no significant “jackpot” effects.

Relative abundance (reported as FPKM and TPM) for each transcript and Unigene were estimated by aligning the reads to the transcriptome assembly using RSEM 1.2.3 [12] (S2 and S3 Files). Best scoring open reading frames were extracted from Trinity transcripts and translated into protein sequences using TransDecoder from the Trinity software package[10]. Protein sequences were annotated using Trinotate from the Trinity package [10] including similarity search against SwissProt database [14] from Uniprot release 2013_08 using BLASTP from BLAST+ 2.2.26 [13], Pfam 27.0 domain [15] predictions in the protein sequences using HMMER 3.0 [27], gene ontology (GO) annotation [16], and orthologous group classification based on eggNOG [17]. A final protein annotation report was generated by Trinotate and included the identifications of Unigenes, Trinity transcripts, and open reading frames with the protein sequences and associated annotations (S4 File). All programs were run with default parameters, except as noted.

### Rat kangaroo transcriptome data sharing

To share the data and make it accessible we have:

1. submitted all raw sequencing reads to the NCBI Sequence Read Archive (SRA; http://www.ncbi.nlm.nih.gov/sra) under accession number SRP055986;
2. included processed data as four Supporting Information files: Trinity assembled transcript sequences in FASTA format (S1 File), isoform abundances (S2 File), Unigene abundances (S3 File) and protein annotations (S4 File);
3. created a web portal (http://dumontlab.ucsf.edu/ratkangaroo.htm) for the rat kangaroo transcriptome where two different tools (described in the main text) allow users to browse Trinity transcripts and find Trinotate-annotated transcript information.

### Transcriptome basic statistics (Table 1)

The total number of clean reads was calculated by subtracting adaptor sequences, ambiguous reads and low quality reads from the total number of raw reads. The Q20 represents the percentage of sequences with a sequencing error rate lower than 1%. The N50 is the length for which the collection of all Trinity transcripts of that length or longer contains half of the sum of the lengths of all Trinity transcripts. Unigenes are distinct loci coding for one or more protein coding Trinity transcripts. Distinct clusters are the number of Unigenes with multiple protein coding Trinity transcripts in the same transcription locus. Distinct singletons are the number of Unigenes with a single protein coding Trinity transcript in their transcription locus.

### Identification of core ribosomal proteins (Table 1)

The "core" ribosomal protein set was defined as all unique protein sequences in the yeast (*Saccharomyces cerevisiae*) 80S ribosome structure (RCSB accession: 4V88) except for suppressor protein STM1. BLASTP of the yeast protein sequences versus the rat kangaroo translated proteins, with an e-value threshold of 1e-3, yielded at least one hit for 64 of the query sequences. An additional protein, L28, was identified via BLASTP of the human protein (GenBank accession NP_001129606.1). TBLASTN of the remaining 10 yeast protein sequences versus the rat kangaroo Trinity transcripts with an e-value threshold of 10^−3^ yielded hits for nine of the query sequences, all in transcripts with no inferred open reading frame (explaining why they were missed by BLASTP). The final protein, L41, is a short, degenerate peptide of 25 amino acids with 10 arginines and seven lysines. Disabling the default SEG complexity filter in TBLASTN and searching with the yeast protein sequence yielded a full-length, 100% identity hit to a rat kangaroo Trinity transcript with no inferred open reading frame. BLASTP and BLASTN were invoked via blastall from NCBI BLAST 2.2.26 using default parameters, except as noted above.

### Assessment of transcriptome coverage by CEGMA (Table 1)

CEGMA version 2.5 (with genewise 2.4.1, hmmer 3.0, blast+ 2.2.26, and geneid v1.4.4.Jan_13_2011) was used to search the transcriptome nucleotide sequences for 248 “ultra conserved” core eukaryotic genes (CEGs) using default parameters. “Complete” orthologs (passing CEGMA's e-value threshold and covering ≥ 70% of the corresponding HMM) were identified for 239 CEGs, and “partial” orthologs (passing CEGMA's e-value but not coverage threshold) were identified for the remaining nine CEGs.

### Selected transcriptome analyses on 25 mitosis genes (Table 2)

Human gene sequences were retrieved from the NCBI GenBank protein database. Each human protein sequence was mapped to the most similar Trinity assembled RNA sequence using the BLAT interface. Multiple isoforms were frequently found for a given gene, with relative abundance of each reflected in the TPM values. To determine whether the most abundant isoform was full-length, the translated protein sequence was searched against the NCBI non-redundant protein database (nr) using BLASTP, restricting to mammalian sequences (axid:40674). The sequence alignments were examined for other marsupials and human to determine whether the sequence was full-length. If so, isoform length and TPM were recorded (Table 2). Otherwise, the above procedure was repeated to find the most abundant full-length isoform. For the most abundant full-length isoform of each gene, the BLASTP search was used to determine the most similar ortholog (determined by BLASTP “Max score”) in the gray short-tailed opossum, Tasmanian devil, and human. The full-length open reading frame of each orthologous protein sequence was aligned with the rat kangaroo full-length open reading frame using the Clustal Omega sequence alignment program to determine sequence identity between species over the entire length of the protein (Table 2). In addition, other isoform sequences for the same rat kangaroo gene were aligned with the determined full-length sequence using Clustal Omega to determine if the gene had multiple full-length isoforms of the protein (Table 2).

### Alignment of PRC1 transcripts from different species (Fig 2)

All full-length rat kangaroo *PRC1* isoforms were found using the method described above. The mRNA sequence was found for each isoform. Full-length human and opossum *PRC1* isoforms and mRNA sequences were found on NCBI. The protein coding regions for these mRNA sequences were aligned using MegAlign (DNASTAR, Madison, WI). The human mRNAs were aligned with human genomic DNA (NCBI) and rat kangaroo and opossum mRNAs were aligned with opossum genomic DNA (NCBI) to find exon lengths and infer splicing sites. Rat kangaroo sequence similarity to opossum was sufficient for alignment of mRNAs to opossum genomic DNA. The length and order of the exons in all three species were identical, which allowed for alignment of all isoforms to each other in Fig 2. For ease of display and comparison, the intron sequence lengths in Fig 2 correspond to the human genomic DNA. Although these intron lengths do not reflect those of opossum and rat kangaroo, the exon lengths and splicing similarities and differences among the transcripts that are shown are correct.

### siRNA design (Fig 3)

Nucleotide sequences to be used for siRNA design were determined using transcript sequences obtained above. Only nucleotide sequences that were conserved across all protein isoforms for our gene of interest and were present in the open reading frame of the gene were used in the design. The nucleotide sequences were evaluated using three siRNA design algorithms (chosen for their ease of use and return of multiple recommendations):

1. Designer of Small Interfering RNA (DSIR) [28,29];
2. Life Technologies BLOCK-iT RNAi Designer [30];
3. InvivoGen siRNA Wizard v3.1 [31].

The length of the siRNA was set to be 19 nucleotides, and siRNAs were selected for further review if they appeared on at least two of the three websites. Selected siRNAs were then screened for off-target effects using the Bowtie Search function on the Maverix browser. siRNAs were discarded if 18 or 19 nucleotides matched with an off-target sequence or if there was a continuous stretch of 16 or more nucleotides that matched with an off-target sequence. Four siRNAs that met these criteria were ordered for knockdown testing in cells.

### PRC1 RNAi in PtK2 cells (Fig 3A)

siRNAs were transfected into PtK2 cells as described elsewhere [6]. Briefly, cells were plated at 25% confluency 24 hours before transfecting. 8 μL of 20 μM siRNA, 4 μL of Oligofectamine (Life Technologies, Carlsbad, CA), and 288 μL Opti-MEM (Life Technologies) were mixed in a microcentrifuge tube, which was flicked every five minutes for 20 minutes to mix. The media on the plated cells was switched to 1.7 mL of OptiMEM + 10% FBS, and the 300 μL of transfection solution was added to each coverslip of cells. Four different siRNAs were transfected into PtK2 cells growing on round 25 mm #1.5 glass coverslips (Warner Instruments # 64–0715, Hamden, CT). After fixing and staining (described below), the coverslips were scored for binucleated cells 48 h post-transfection as a readout of *PRC1* knockdown efficiency. The siRNA with the highest percentage of binucleated cells (siPRC1-A: 5’-GGACTGAGGTTGTCAAGAA-3’) was the sole one used for further validation of the knockdown.

### Immunoblotting (Fig 3B)

PtK2 cells were plated at 25% confluency and transfected 24 h later with siPRC1-A or Oligofectamine only as a control. Transfections were performed as described above. Then, 48 h post-transfection, the cells were lysed, and the lysate was run on a protein gel and transferred to a nitrocellulose membrane and probed with mouse anti-PRC1 (1:1,000, BioLegend #629001, San Diego, CA) and anti-tubulin DM1α (1:5,000, Sigma #T6199, St. Louis, MO) as a control. The blots were probed with goat anti-mouse IgG-HRP (1:10,000, Santa Cruz Biotechnology, Inc. #sc-2055, Santa Cruz, CA). The blots were exposed with SuperSignal West Femto Maximum Sensitivity Substrate (Thermo Scientific #34095) and imaged with Bio-Rad (Hercules, CA) ChemiDoc XRS+ system. Images were quantified using ImageJ 1.48u (NIH).

### Immunofluorescence and quantification of binucleated cells (Fig 3C–3E)

To look at mock control and siPRC1-A-treated cells (Fig 3D and 3E), cells were fixed 48 h post-transfection in 95% methanol with 5 mM EGTA for 3 min. The following antibodies and dyes were used: mouse anti-PRC1 (1:100, BioLegend #629001), human anti-centromere (1:25, Antibodies Incorporated #15–234, Davis, CA), rabbit anti-α-tubulin (1:200, Abcam #ab18251, Cambridge, United Kingdom), Hoescht 33342 (Sigma-Aldrich), goat anti-mouse-488 (1:500, Life Technologies #A11001), goat anti-human-594 (1:500, Life Technologies # A11014), and goat anti-rabbit-647 (1:500, Life Technologies #A21244). Images were captured at 100X magnification on a spinning disk confocal inverted microscope described elsewhere [32]. Briefly, interphase cells were imaged with exposure times of 50–1,000 ms with 405, 488, 561, and 647 nm light. Dividing cells were imaged with exposure times of 50–200 ms with 405, 488, 561, and 647 nm light light. To count binucleated cells (Fig 3C), the above cells for both mock control and siPRC1-A conditions were imaged at 20X magnification (on a Zeiss (Oberkochenhttp://en.wikipedia.org/wiki/Oberkochen, Germany) AxioPlan2 epifluorescence microscope). The total number of interphase cells was counted for all images in both conditions, and the number of binucleated interphase cells was counted and reported as a percentage of total cells. Hoechst DNA staining was used to determine whether the cells were mono- or binucleated.

### Live cell imaging of PRC1 depleted cells (Fig 3F)

PtK2 cells were imaged 72 h post-transfection. Cells were imaged at mitosis by phase contrast and 488 nm laser light every 2 min on a spinning disk confocal inverted microscope described elsewhere [32]. Briefly, cells were imaged with 50 ms exposures with 488 nm light and 400 ms exposures with transmitted light. Cells were imaged at 30°C and 5% CO2 in a Tokai Hit (Fujinomiya, Japan) PLAM environmental chamber, and using the Perfect Focus System (Nikon, Tokyo, Japan). Mitotic cells were imaged for several hours until it was clear that cytokinesis had either successfully completed or failed.

## Supporting Information

S1 File Trinity assembled transcript sequences. cDNA sequences of Trinity assembled transcripts in FASTA format.
(BZ2)

S2 File Isoform abundances. RSEM estimates of transcript isoform abundances in tab delimited format. See RSEM documentation for column descriptions.
(BZ2)

S3 File Unigene abundances. RSEM estimates of Unigene abundances in tab delimited format. See RSEM documentation for column descriptions.
(BZ2)

S4 File Protein annotations. Trinotate annotation of translated open reading frames for all transcript isoforms in tab delimited format. Missing values are indicated with a dot (.). Columns: -component: Unigene unique ID. -trans_derived: Location of annotated open reading frame, as isoform:start-stop(strand). -prot_id: Unique ID of translated protein sequence. -TopBlastHit: Top BLASTP hit for annotated protein. Fields are backtick (`) delimited and are: GenBank ID, GenBank accession, HSP cooridinates relative to query and subject, %ID, E-value, gene names, and taxonomic lineage of the subject. -Pfam: hmmer-derived Pfam domain hits, backtick (`) delimited. Fields for each Pfam hit are caret (^) delimited and are: Pfam ID, Pfam name, Pfam description, domain location in protein, E-value. -eggnog: eggNOG hit. Fields are caret (^) delimited and are: eggNOG 3.0 ID, eggNOG description. -gene_ontology: GO annotations, backtick (`) delimited. Fields for each hit are caret (^) delimited and are: GO ID, GO aspect, GO term. -prot_seq: amino acid sequence of translated open reading frame.
(BZ2)
